# Visual Search for Circumscribed Interests in Autism Is Similar to That of Neurotypical Individuals

**DOI:** 10.3389/fpsyg.2020.582074

**Published:** 2020-10-21

**Authors:** Benjamin M. Silver, Mary M. Conte, Jonathan D. Victor, Rebecca M. Jones

**Affiliations:** ^1^Department of Psychology, Columbia University in the City of New York, New York, NY, United States; ^2^Feil Family Brain and Mind Research Institute, NewYork-Presbyterian Hospital, Weill Cornell Medicine, New York, NY, United States; ^3^Department of Neurology, NewYork-Presbyterian Hospital, Weill Cornell Medicine, New York, NY, United States; ^4^The Sackler Institute for Developmental Psychobiology, NewYork-Presbyterian Hospital, Weill Cornell Medicine, New York, NY, United States

**Keywords:** autism spectrum disorder, visual processing, serial processing, parallel processing, circumscribed interests, visual search

## Abstract

Intense interests are a core symptom of autism spectrum disorders (ASD) and can be all-encompassing for affected individuals. This observation raises the hypothesis that intense interests in ASD are related to pervasive changes in visual processing for objects within that category, including visual search. We assayed visual processing with two novel tasks, targeting category search and exemplar search. For each task, three kinds of stimuli were used: faces, houses, and images personalized to each participant’s interest. 25 children and adults with ASD were compared to 25 neurotypical (NT) children and adults. We found no differences in either visual search task between ASD and NT controls for interests. Thus, pervasive alterations in perception are not likely to account for ASD behavioral symptoms.

## Introduction

Intense interests are a common symptom of autism spectrum disorder (ASD) (South et al., 2005; Turner-Brown et al., 2011; Klin et al., 2013) and are a specific kind of Restricted and Repetitive Behavior (RRB) (American Psychiatric Association, 2013). The current study focuses on the possible relationship between intense interests and visual processing. Interests are highly motivating for individuals with ASD (Winter-Messiers et al., 2008), and when incorporated into therapy, interests can have a positive effect on ASD clinical outcomes and academic achievement (Boyd et al., 2007; Koegel et al., 2012, 2013; Kryzak et al., 2013; Gunn and Delafield-Butt, 2015; Harrop et al., 2019). However, interests can also be detrimental to daily functioning by interfering in day-to-day activities and social interactions (Klin et al., 2013).

One possible link between interests and visual processing is that ASD symptoms associated with intense interests may produce abnormal visual perception for images related to interests, similarly to how experts demonstrate enhanced visual pocessing for their category of expertise (Gauthier et al., 2000). Alternatively, individuals with ASD may have a primary underlying alteration in the visual system, which leads to intense interests. For example, while neurotypical (NT) controls are hardwired to rapidly process faces and quickly search for faces (Bruce and Humphreys, 1994; Tong and Nakayama, 1999), it is possible that individuals with ASD may respond more quickly to intense interests and show visual expertise for interests similar to how NT controls process faces. We explore these possibilities to better understand the phenomenon of intense interests in ASD.

In order to understand the possible mechanisms of visual expertise for intense interests in ASD, it is important to provide an overview of the forms that visual expertise can take in typical development. For example, visual expertise for faces is widely studied. Face-to-face interactions are the foundation of daily functioning and it is thought that starting early in life, neurotypical individuals are particularly attuned to faces (Mondloch et al., 1999). Evidence for visual expertise for faces comes from a robust behavioral literature (Treisman and Gelade, 1980; Schwarzer, 2000; Hershler and Hochstein, 2005) as well as from functional MRI (fMRI) work, and is supported by neurophysiologic studies in non-human primates (Tsao et al., 2006). Faces uniquely activate a distributed network in the brain that includes the fusiform gyrus (FFA) (Kanwisher et al., 1997; O’Toole et al., 2005), as well as other visual processing areas, including the occipital face area (Anderson et al., 2000).

While visual expertise for faces is pervasive, visual expertise for classes other than faces may also be present in neurotypical individuals (Wood, 1999). For example, a hallmark study by Chase and Simon (1973) demonstrated that chess experts are better at remembering structured chessboard arrangements than novices. More recent eye-tracking studies have shown chess experts make fewer and more holistic fixations when looking at non-random chess board arrangements (Reingold et al., 2001). Visual expertise can also be developed for individuals who spend several hours a day playing hockey (Canal Bruland et al., 2010), video games (Latham et al., 2013), or badminton (Abernethy and Russell, 1987), as well as in certain occupational fields such as medical diagnostics (Crowley et al., 2003) and air traffic control (Van Meeuwen et al., 2014). In a laboratory setting, visual experts have improved short-term memory for their object of expertise (Curby et al., 2009) and have higher signal detection scores (d-prime) when matching different images of the same exemplar object (for example, matching car models from different years) (Gauthier et al., 2000). In all of the above circumstances, individuals demonstrate enhanced visual search and selective attention for their (non-social) expertise.

Visual processing studies in ASD have shown perceptual differences for both social stimuli (faces) as well as non-social stimuli (objects), with some evidence that perception of non-social stimuli in ASD can resemble perception for social stimuli in an NT population (Sasson et al., 2008). Individuals with ASD prefer to look at objects over faces and look at faces less than NT controls (Unruh et al., 2016). These preferences for non-social objects may be present in children diagnosed with ASD as young as two (Klin et al., 2009). Lastly, fMRI studies have demonstrated that individuals with ASD recruit the FFA for non-social objects of interest more than NT controls (Foss-Feig et al., 2016), suggesting that individuals with ASD process interests similarly to how NT individuals process faces. There is a large literature around early visual processing in ASD (Dakin and Frith, 2005; Van der Hallen et al., 2015), with conflicting results depending on what aspect of visual processing is probed. Studies of early visual processing in ASD show enhanced visual processing for fine details, both during visual search (O’Riordan et al., 2001) and in luminance contrast (Luc et al., 2011), but also find deficits in other areas, such as binocular rivalry (Robertson et al., 2013), mental imagery (Marothi et al., 2019), and motion perception (Milne et al., 2002; Bertone et al., 2003), with some work demonstrating this deficit can be found as early as the primary visual cortex (Robertson et al., 2014).

Visual search is a specific type of visual processing that is closely tied to spatial attention (Wolfe, 2015). Visual search tasks involve locating a target item amongst a set of distractor items of variable set size. Visual search is also flexible, with adjusted strategies based on set size and complexity (Wolfe et al., 1992) and separable from working memory (Horowitz and Wolfe, 1998). In NT individuals, visual search tasks involving faces demonstrate high efficiency in search compared to other object types (Bruce, 1986), even for faces that are only viewed for a brief period of time (Diamond and Carey, 1986).

In one common visual search paradigm, participants must have *categorical* knowledge of an object in a specific category, or knowledge about how the object is different from objects in other categories. In this paradigm, participants search for images of a particular category (butterflies or cars, for example) amongst an array of unrelated distractor images, such as animals or articles of clothing. Experts in a particular category have higher search efficiency on that category than non-experts (Hershler and Hochstein, 2009; Golan et al., 2014). In a contrasting type of visual search paradigm, participants must have *exemplar* knowledge, meaning that they must be able to pick out an image that is consistent with a category of distractor images. For the bird category of the Vanderbilt Expertise Test, for example, participants spend several seconds viewing a group of images of birds, followed by a second set of novel bird images in which the participant must find an image that depicts a matching species from the first group (McGugin et al., 2012). These two paradigms differ in the distinction (category vs. exemplar) that must be picked out during visual search. Furthermore, category and exemplar search differ in complexity and difficulty, with category search requiring the knowledge of early visual components of a category, and exemplar search requiring broader knowledge about specific instantiations of a category.

As visual expertise is not as a monolithic process, consideration must be given to the origins of alterations in the visual pathway. There are two overall ways that visual expertise and intense interests may be related in ASD: intense, non-social interests may alter visual experience, leading to expertise, or alterations in the normal development of the visual system may result in object categories taking over circuitry that is typically specialized for faces, leading to the development of intense, non-social interests. Given the alterations of spatial attention in ASD (Townsend et al., 2001; Sokhadze et al., 2016), visual search is a particularly relevant method for understanding visual processing in ASD. Visual search tasks readily measure certain aspects of visual expertise, and can test whether intense interests are indeed associated with a shift in this domain. Prior work on visual search in ASD suggests enhanced visual search abilities with neutral object stimuli such as shapes, letters, or common objects, as compared to NT controls, with faster reaction times and higher accuracy levels (Joseph et al., 2009; Kaldy et al., 2016). It is unknown whether individuals with ASD will demonstrate enhanced visual search capabilities for individualized interests.

The present study tested visual expertise for intense interests in children and adults with ASD compared to controls with two novel visual search paradigms that distinguished category vs. exemplar search abilities (Jonides and Gleitman, 1972; Smilek et al., 2006). Building upon prior visual expertise paradigms, personalized images of each participant’s interest or hobby were compared to images of faces and houses. Given the work that demonstrates that non-social objects are processed atypically in ASD, and that categories of expertise can be accompanied by enhanced visual search abilities, we hypothesized that visual search abilities to intense interests in ASD would be enhanced in both the category and exemplar tasks, resulting in reduced reaction times or possibly greater search efficiency. Enhanced performance in either of these tasks would suggest that intense interests in ASD are a visual atypicality. Inclusion of both a category and an exemplar task, which draw on different visual search processes, allowed us to be more specific in our diagnosis of the origin of intense interests and to increase our ability to identify a visual-based performance difference. We also predicted enhanced visual search skills for faces in NT controls would not be observed in individuals with ASD.

Finally, we mention another advantage of studying search: as mentioned above, in NT subjects, search tasks involving faces are substantially more efficient than search tasks for other object categories (Bruce, 1986; Diamond and Carey, 1986). As this is a robust and consistent finding in NT subjects, we reasoned (and the statistical analyses below confirm) that a modest subject pool has high power in identifying whether this characteristic of search is substantially altered in ASD subjects.

## Materials and Methods

### Participants

Fifty participants (children ages 5–16 and adults ages 18–30) completed one of two tasks–a category search task and an exemplar search task, both described in detail below. 32 participants (17 ASD, 18 children) completed the category search task and 30 participants (16 ASD, 18 children) completed the exemplar search task; 12 participants completed both–eight ASD (five children, three adults) and four NT (four children, zero adults). Two of the 17 ASD participants who completed the category task were excluded from analyses due to incomplete data. Of the adults with ASD, five were their own legal guardian, and four had a caregiver as their guardian. Of the children with ASD, all attended school full-time. Participants with ASD (six females) were recruited through the Center for Autism and the Developing Brain (CADB) in White Plains, NY, United States. Neurotypical (NT) controls (nine females) were recruited through the Sackler Institute for Developmental Psychobiology in Manhattan, NY and through the local New York City community. Informed written consent (assent from minors, consent from caregivers) was obtained from all participants and the study protocol was approved by the Weill Cornell Medicine Institutional Review Board.

### Phone Interview

One to two weeks before a participant’s in-person testing, a 5-min telephone interview was conducted to assess participants’ primary interests. For participants under 13 years old, the interview was conducted with a caregiver. Only two participants in the child group did not fall into this category. The participant (or caregiver) was asked to name three activities or topics that he or she enjoyed doing or thinking/learning/talking about. For each interest, the participant was asked to elaborate on specific aspects of the interest that he or she liked, to indicate how long he or she has had this interest, and whether the interest had changed or developed over time. The participant was also asked to specify which of the three interests were most prominent at the time of the interview. The questions were designed to target the specific aspects of the topic or activity that was most appealing in order to identify stimuli to be used in the tasks. Multiple interests were queried in case the most prominent interest could not be easily represented visually (such as listening to music). ASD participants and caregivers consistently reported interests that were more intense and more specific (as indicated by statements such as “he watches the same movie over and over again,” or a preference for particular movies or episodes in a series as opposed to the series as a whole) than those reported by NT caregivers. All answers were recorded on paper and stored with the participant’s data folder.

### Autism Assessments and Cognitive Testing

Participants with ASD received a diagnosis from a trained clinician at CADB using Module three or four of the Autism Diagnostic Observation Schedule (ADOS) (Lord et al., 2012) prior to participation. Total calibrated severity scores (CSS) were generated from the ADOS as well as for Social Affect (SA) and RRB (Hus et al., 2014). NT participants under 18 years old were screened for ASD symptoms with the Social Communication Questionnaire (SCQ-Lifetime) (Rutter et al., 2003), and participants 18 years old and older were screened with the Autism Spectrum Quotient (AQ) (Baron-Cohen et al., 2001). Participants were deemed eligible if they had scores under 15 on the SCQ and scores under 32 on the AQ. Two participants were missing SCQ scores, and in these cases the Social Responsiveness Scale-2 (SRS-2) (Constantino and Gruber, 2012) was used, with a cutoff score of 70. One NT participant was excluded from category task analyses based on their SCQ score. Cognitive skills were measured in participants under 16 years of age with the Differential Abilities Scale-II (school age) (DAS) (Elliott, 2007), and participants 16 years old and older completed the Wechsler Adult Intelligence Scale (WAIS-IV) (Wechsler, 2008). Standard scores for verbal IQ (VIQ) and non-verbal IQ (NVIQ) were derived from the DAS-II or WAIS-IV (see Table 1 for full demographic information).

**TABLE 1:** Participant demographics.

	ASD Children		ASD Adults		NT Children		NT Adults	
	Category	Exemplar	Category	Exemplar	Category	Exemplar	Category	Exemplar
Age–Mean (Range)	10.73 (5.75–15.83)	12.09 (7–16.17)	25.53 (20.08–30.33)	24.96 (21.33–31.25)	9.92 (6.25–12)	9.63 (6.08–13.08)	22.58 (19.83–27.67)	25.15 (21.58–29.33)
# of Females/Males	2/9	1/9	4/2	3/3	2/8	1/7	2/3	4/2
AQ–Mean (SD); Range	N/A	N/A	N/A	N/A	N/A	N/A	13.2 (4.09); 7–18	22.5 (3.62); 18–27
SCQ–Mean (SD); Range	N/A	N/A	N/A	N/A	5.13 (5.33); 1–16	4.13 (3.31); 1–9	N/A	N/A
VIQ–Mean (SD); Range	98.81 (19.5); 71–143	103.10 (20); 71–143	104 (8.74); 95–118	101 (5.33); 95–108	112.30 (14.2); 90–136	115.75 (17); 86–136	124.20 (13.7); 110–145	113.17 (10.5); 102–127
NVIQ–Mean (SD); Range	96.81 (15.3); 77–131	101.30 (13.6); 84–131	98.5 (13.5); 81–119	95.83 (8.64); 81–104	108.80 (18.2); 89–149	108.13 (14.3); 80–121	116.60 (8.65); 105–127	106.67 (6.98); 98–117
ADOS CSS–Mean (SD); Range	7.20 (1.99); 4–10	7.50 (1.96); 4–10	7.67 (0.816); 7–9	8 (0.894); 7–9	N/A	N/A	N/A	N/A

### Interest Assessments

At the in-person visit, participants (or caregivers) completed a questionnaire about the participant’s topic or activity of interest identified through the phone interview. There was a child version administered to caregivers and an adult version completed via self-report. The questionnaire asked the caregiver or participant to specify what they knew about or did involving their topic or activity of interest, how much it interfered with day-to-day activities such as spending time with friends/family and going to school/work, and to indicate the duration of their interest on a 1–5 scale (1 = less than 6 months, 5 = over 5 years). From the questionnaire, two scores were derived: an “Interference” measurement, defined as the average rating on the questions concerned with how much the interest took time from activities related to friends, family, school and/or work, and a “Current Time” measurement, defined as the average rating on questions concerned with the amount of time spent on the interest on a day-to-day basis. On the child version, scores ranged from 1 to 3 (less than 25% of the time, 25–75% of the time, over 75% of the time), and on the adult version, scores ranged from 1 to 5 (1 = strongly disagree, 3 = neither agree nor disagree, 5 = strongly agree), and were converted to a one to three scale to match the child version. While using two different questionnaires may make it more difficult to compare scores, each version of the questionnaire was designed to be completed by a specific age range, and thus differentiating them was necessary.

### Category Search Task

**FIGURE 1:**
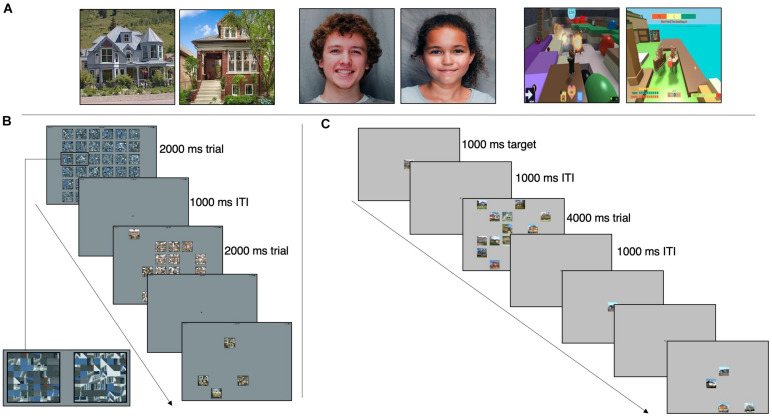
**(A)** Example stimuli from the houses, faces, and interests categories (left to right). House stimuli are from Konkle et al. (2010) and face stimuli are from Meuwissen et al. (2017). **(B)** Category Search Task. Participants instructed to find the unscrambled image. Three example trials displayed. 36-image array presented for 2,000 ms with 35 scrambled images and one target, a 1,000-ms ITI, followed by a 16-image array and ITI, and lastly a four-image array. Box in first image of sequence is enlarged to show example of scrambled images. **(C)** Exemplar Search Task. Participants instructed to find the target image. Two example trials displayed. Target presented for 1,000 ms, a 1,000-ms ITI and a 16-image array with 15 distractors and the target image. A 1,000-ms ITI separates this trial from the next target presentation, which is part of a four-image array trial.

This task, presented on an iPad (Model number: A1822, 9.4 in. × 6.6 in.), made use of three categories: Houses, Faces, and Interests (Figure 1A). Stimuli for Houses were 108 unique photos of houses gathered from the internet and a stimulus set by Konkle et al. (2010). Stimuli for Faces were 108 unique full-face photos of child and adult faces from the Developmental Emotional Faces Stimulus Set by Meuwissen et al. (2017). While this stimulus set has not been previously used in an ASD population, it was chosen because the age range of faces (8–30 years old) was similar to the age range of the participants. To avoid possible confounds due to differences in emotional processing between NT and ASD individuals, only happy faces were used. The Interests category was individually tailored for each participant based on the phone interview; for example, a participant who indicated on the phone that his/her primary interest was the video game “Minecraft” saw screenshots from the video game (see Figure 1A). Interests stimuli were 108 unique photos of the participant’s interest gathered from the internet (see Supplementary Table S1 for a list of interests). While some of the Interests stimuli were related to people, such as TV shows or movies, and therefore contained faces, none of the images displayed faces in a prominent manner, thus distinguishing them from the large, centered, and in-focus faces in the Faces condition. All stimuli were resized to 256 × 256 pixels using MATLAB software.

There were three practice trials and 108 test trials per category. A trial consisted of either 4, 16, or 36 images in a random array for 2,000 ms, followed by a central fixation cross for 1,000 ms. The trial duration of 2,000 ms was used based on the performance of pilot subjects. There were 36 trials for each array size. In each trial, images were presented in a random array (see Figure 1B). One image, the target, was intact; the distractor images were created by scrambling the target image based on a random repositioning of an 8 × 8 grid of sub-blocks. Scrambled images were used as distractors as opposed to other-category images (as used in Golan et al., 2014 and Hershler and Hochstein, 2009) to avoid potential visual processing differences in ASD for different categories of objects, which could have confounded interpretation of a positive result. In addition, some categories of objects are more similar than others; scrambling images allowed the distractor difficulty to be standardized. The same image size was used for all three array sizes. A constant image size but variable array size was chosen so that the slope of RT vs. array size could be measured without a size confound. This is a standard approach in studies of visual search (Tong and Nakayama, 1999; Smilek et al., 2006). Images were scrambled using MATLAB.

Participants were instructed to find and touch the target as quickly as possible. The position of the target in the array was randomized, but the average target position across all trials was the center of the array. Trial order was randomized, and blocks for each category were run in random order. Participant accuracy (correct or incorrect) and reaction times were recorded for each trial.

### Exemplar Search Task

The exemplar task, presented on the same iPad from the category task, consisted of the same three categories (Houses, Faces, and Interests) as the category task. Image size was the same as in the category task. Each category contained 54 trials. A trial consisted of a single target image presented at the center of the display for 1,000 ms, a 1,000 ms crosshair, and then the target and either 3, 8, or 15 distractors in a random array for 4,000 ms (a longer search time than that of the category task due to the increased difficulty of this task). In contrast to the category search task, distractor images were not scrambled images but were different examples drawn from the same category as the target (see Figure 1C). There were 18 trials for each array size. Participants were instructed to find and touch the target as quickly as possible. Each trial’s target was unique, but the distractors repeated between trials. Trial order and category order were randomized. Participant accuracy (correct or incorrect) and reaction times were recorded for each trial.

### Data Analysis

Primary analyses were identical for both tasks. Accuracies and average reaction times (RTs) were calculated for each array size and category. Accuracy was defined as number of correct trials out of the total number of trials for each array size. For each category, a slope (milliseconds/item) was calculated from the average RTs, determined from the regression (least-squares) of average RT vs. array size. Trials in which no response was registered in the allotted time (2,000 ms for the category task and 4,000 ms for the exemplar task) were counted as misses in the accuracy measurement and were excluded from all RT analyses.

To assess the effects on accuracy and RTs, a 3 (category) × 3 (array size) × 2 (diagnosis) × 2 (age group) ANOVA was performed for both measures. To assess the effects on slope, a 3 (category) × 2 (diagnosis) × 2 (age group) ANOVA was performed. Age of participants was binarized into two groups, children and adults. *p*-values from the ANOVA are reported without correction for multiple comparisons, as our main focus is on whether there is an interaction between diagnosis and category (a single comparison for each ANOVA), and we wanted to maximize the sensitivity to detect such interactions. Significant main effects and interactions were interrogated with *post hoc t*-tests. In the body of the “Results” section, the *F*-values and *p*-values are provided for significant effects and interactions, and only the *p*-value is provided for non-significant effects and interactions. The full statistics for all tests can be found in Tables 2, 3.

**TABLE 2A:** Category task statistics.

		Accuracy		Reaction Time		Slope		
		*F*	*P*	*F*	*p*	*F*	*p*	DoF
Main Effects	Dx	0.108	0.745	1.168	0.290	0.265	0.611	(1, 25)
	Age	3.334	0.080	7.907	0.009	12.647	0.002	(1, 25)
	Category	12.678	< 0.001	156.534	< 0.001	44.520	< 0.001	(2,50)
	Array Size	55.565	< 0.001	149.330	< 0.001			(2, 50)
Two-Way Interactions	Dx × Age	0.075	0.787	0.979	0.332	1.092	0.306	(1, 25)
	Dx × Category	0.980	0.382	0.347	0.709	0.074	0.929	(2, 50)
	Age × Category	2.492	0.002	0.250	0.780	12.533	< 0.001	(2,50)
	Dx × Array Size	0.736	0.484	0.547	0.582			(2, 50)
	Age × Array Size	3.594	0.035	7.504	0.001			(2, 50)
	Category × Array Size	25.836	< 0.001	26.669	< 0.001			(4, 100)
Three-Way Interactions	Dx × Age × Category	0.135	0.874	1.099	0.341	0.361	0.699	(2, 50)
	Dx × Age × Array Size	1.103	0.340	0.642	0.430			(2, 50)
	Dx × Category × Array Size	0.187	0.945	0.472	0.757			(4, 100)
	Age × Category × Array Size	0.794	0.532	7.719	< 0.001			(4, 100)

*Dx, Diagnosis; DoF, Degrees of Freedom.*

**TABLE 2B:** Category task statistics (*Post hoc* analyses).

		Accuracy		Reaction Time		Slope	
	Comparison	*T*	*p*	*t*	*p*	*t*	*p*
Category	Faces vs. Houses	3.401	0.002	–14.313	< 0.001	–6.658	< 0.001
	Faces vs. Interests	6.713	< 0.001	–18.217	< 0.001	–5.437	< 0.001
	Houses vs. Interests	1.269	0.215	–4.108	< 0.001	–0.154	0.879
Array Size	4 vs. 16	2.313	0.028	–3.133	0.004		
	4 vs. 36	8.220	< 0.001	–12.768	< 0.001		
	16 vs. 36	10.091	< 0.001	–11.257	< 0.001		

*Degrees of Freedom for all comparisons = 28.*

**TABLE 3A:** Exemplar task statistics.

		Accuracy		Reaction Time		Slope		
		*F*	*P*	*F*	*p*	*F*	*p*	DoF
Main Effects	Dx	0.018	0.895	0.811	0.376	0.352	0.558	(1, 25)
	Age	11.097	0.003	13.420	< 0.001	1.229	0.278	(1, 25)
	Category	12.598	< 0.001	1.839	0.169	1.503	0.232	(2, 50)
	Array Size	73.139	< 0.001	114.863	< 0.001			(2, 50)
Two-Way Interactions	Dx × Age	1.245	0.275	2.702	0.113	0.021	0.885	(1, 25)
	Dx × Category	0.606	0.550	0.113	0.894	0.105	0.900	(2, 50)
	Age × Category	1.025	0.366	1.315	0.278	0.185	0.832	(2, 50)
	Dx × Array Size	0.136	0.873	1.210	0.307			(2, 50)
	Age × Array Size	3.717	0.031	1.247	0.296			(2, 50)
	Category × Array Size	2.488	0.048	1.174	0.327			(4, 100)
Three-Way Interactions	Dx × Age × Category	0.699	0.502	0.603	0.551	0.003	0.997	(2, 50)
	Dx × Age × Array Size	1.782	0.179	0.016	0.984			(2, 50)
	Dx × Category × Array Size	1.134	0.345	0.092	0.985			(4, 100)
	Age × Category × Array Size	0.554	0.697	0.288	0.885			(4, 100)

*Dx: Diagnosis; DoF: Degrees of Freedom.*

**TABLE 3B:** Exemplar task statistics (*Post hoc* analyses).

		Accuracy		Reaction Time		Slope	
	Comparison	*t*	*P*	*T*	*p*	*t*	*p*
Category	Faces vs. Houses	2.392	0.024	0.532	0.599	0.691	0.495
	Interests vs. Houses	5.002	< 0.001	–1.391	0.175	–1.089	0.285
	Interests vs. Faces	3.338	0.002	–2.124	0.042	–1.971	0.058
Array Size	4 vs. 9	4.121	< 0.001	–14.300	< 0.001		
	4 vs. 16	9.864	< 0.001	–13.225	< 0.001		
	9 vs. 16	9.857	< 0.001	–6.334	< 0.001		

*Degrees of Freedom for all comparisons = 28.*

## Results

### Questionnaires

On the interest questionnaire, adults with ASD scored higher on Current Time than NT adults (*t*(15) = 3.972, *p* = 0.001), but there was no difference in Interference (*t*(15) = –0.763, *p* = 0.458). In the child group, there was a trend of a difference in Interference (*t*(26) = 1.887, *p* = 0.070), but no difference in Current Time (*t*(26) = 1.587, *p* = 0.125), likely due to the limited range in response options on the child version compared with the adult version.

Verbal and non-verbal IQ scores were significantly different or nearly so for both the category task and the exemplar task (VIQ category task: *t*(30) = –2.822, *p* = 0.008; NVIQ category task: *t*(30) = –2.633, *p* = 0.013; VIQ exemplar task: *t*(28) = –2.242, *p* = 0.033; NVIQ category task: *t*(28) = –1.929, *p* = 0.064), with ASD participants demonstrating lower scores than NT participants. However, with age and diagnosis as regressors, neither slope, accuracy, nor RT were significantly correlated with IQs on either the category task (*p*’s > 0.318) or the exemplar task (*p*’s > 0.088).

There was a significant difference between AQ scores of participants in the category task vs. the exemplar task (*t*(9) = –4.006, *p* = 0.003).

### Category Task

#### Accuracy

**FIGURE 2:**
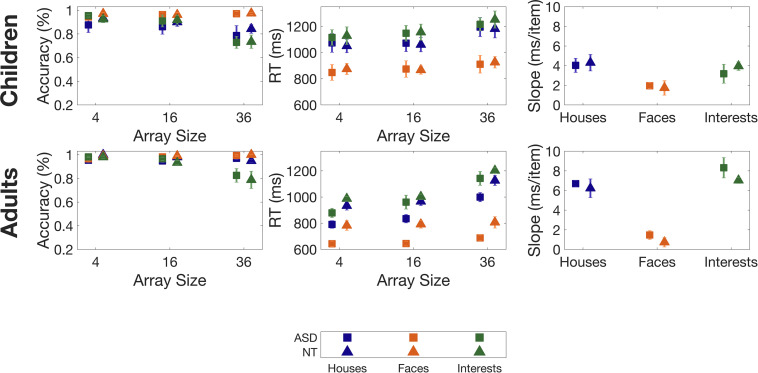
Accuracies, Reaction Times (RTs) and slopes for the category task, for children (top panels) and adults (bottom panels). Slopes are determined from a least-squares regression of RT vs. array size. Error bars indicate 1 Standard Error of the Mean.

Overall accuracy on the task was high, on average 92%. Accuracy for Faces was highest, followed by Houses and Interests (see Table 2 for full statistics, including *F*-values for non-significant comparisons). As expected, accuracy was highest for the smallest array size, and decreased as array size increased. There was a trend of a main effect of age with adults having an overall higher accuracy than children (Adults Mean: 96%; Children Mean: 90%, *p* = 0.08) (see Figure 2). However, there was no main effect of diagnosis on accuracy (*p* = 0.745), and no interaction between diagnosis and category (*p* = 0.382).

#### Reaction Time

Participants’ reaction times were different for each category (*F*(2,50) = 156.534, *p* < 0.001) with faster RTs for Faces than for Houses and for Interests, and faster RTs for Houses than for Interests. Reaction times were also influenced by array size (*F*(2,50) = 149.330, *p* < 0.001) with faster RTs for smaller array sizes. An interaction between array size and category (*F*(4,100) = 26.669, *p* < 0.001) was explained by less change in RTs for Faces across array size compared to Houses and Interests.

Adults had faster RTs than children (*F*(1,25) = 7.907, *p* = 0.009). An interaction between array size and age (*F*(2,50) = 7.504, *p* = 0.001) was explained by a larger gap in RTs between adults and children on smaller array sizes than on larger ones (see Figure 2). However, there was no significant main effect of diagnosis on RTs (*p* = 0.290), and no interaction between diagnosis and category (*p* = 0.709).

#### Slope

Slope changed with category (*F*(2,50) = 44.520, *p* < 0.001) as participants had lower slopes for Faces relative to Houses and Interests. There was no difference in slope between Houses and Interests.

There was a main effect of age (*F*(1,25) = 12.647, *p* = 0.002). While adults overall had lower RTs than children (see above), adults overall had higher slopes than children (see Figure 2). Given that the slope is a value derived from the average RT values for each array size, this suggests that on Houses and Interests, while children performed worse than adults on smaller array sizes, as array size grew the age-related performance gap shrunk. There was no effect of diagnosis (*p* = 0.611) on slope, and no interaction between diagnosis and category (*p* = 0.929).

### Exemplar Task

#### Accuracy

Overall accuracy on the task was 79%. Accuracy was impacted by category (*F*(2,50) = 12.598, *p* < 0.001) and was higher for Interests than for Faces, and was higher for Faces than for Houses (see Table 3 for full statistics). As expected, array size impacted accuracy (*F*(2,50) = 73.139, *p* < 0.001), with a decrease in accuracy as array size grew.

**FIGURE 3:**
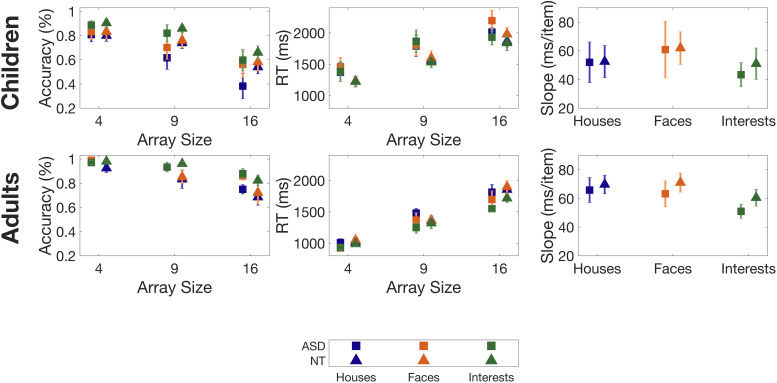
Accuracies, Reaction Times (RTs) and slopes for the exemplar task, for children (top panels) and adults (bottom panels). Slopes are determined from a least-squares regression of RT vs. array size. Error bars indicate 1 Standard Error of the Mean.

Adults had higher accuracy than children (*F*(1,25) = 11.097, *p* = 0.003). While accuracy for some children was quite low (below 60%), all participants exhibited the same decrease in accuracy as array size increased, suggesting that the low accuracy was a result of an overall increase in task difficulty, rather than a misunderstanding of task instructions (see Figure 3). There was no effect of diagnosis on accuracy (*p* = 0.895), and there was no interaction between diagnosis and category (*p* = 0.550).

#### Reaction Time

Category did not impact RTs (*p* = 0.169). RTs were impacted by array size (*F*(2,50) = 114.863, *p* < 0.001), with RTs increasing as array size increased.

Adults had lower RTs than children (*F*(1,25) = 13.42, *p* = 0.001) (see Figure 3). There was no significant effect of diagnosis (*p* = 0.380) and no significant interaction between diagnosis and category (*p* = 0.894).

#### Slope

Category (*p* = 0.230) and age (*p* = 0.280) did not influence slopes (see Figure 3). The lack of difference in age, paired with the distinct differences in age on RTs and accuracies, suggests that while children performed worse on the task than adults overall, both groups were affected by the increase in array sizes equally. There was also no main effect of diagnosis (*p* = 0.558), and there was no interaction between diagnosis and category (*p* = 0.900).

### Power Analyses

As our findings did not reveal a significant difference in search performance in ASD participants vs. NT controls, we undertook power analyses to determine the likelihood that, if substantial differences were present, they would have been detected. Power analyses are summarized in Table 4 and detailed below. Briefly, owing to the consistency of findings in NT subjects, the category task has power of >98% in revealing either an absence of a greater efficiency for Faces, or a reversal of efficiency between Faces and Interests. The exemplar task was underpowered for identifying an absence of differential efficiency for Faces (17%), and had a power of approximately 70% for revealing a reversal, but nevertheless adds to the overall power of the study.

**TABLE 4A:** Power Analysis, scenario (i): no difference between faces and interests in ASD subjects.

		Number of significant ANOVAs out of 1,000			
		Accuracy	Reaction Time	Slope	Overall
Dx × Category ANOVA	Category Task	481	956	376	987
	Exemplar Task	76	50	56	171

**TABLE 4B:** Power Analysis, scenario (ii): reversed difference between faces and interests in ASD subjects.

		Number of significant ANOVAs out of 1,000			
		Accuracy	Reaction Time	Slope	Overall
Dx × Category ANOVA	Category Task	1,000	1,000	999	1,000
	Exemplar Task	581	163	176	714

The power analyses were conducted via a bootstrap, a standard procedure for determining study power *post hoc* (Efron and Tibshirani, 1998; Walters, 2004). We considered two hypothetical scenarios in which the well-known specialized processing for faces expected in NT subjects (and confirmed here) might be altered in a way that could account for ASD symptomatology. In scenario (i), individuals with ASD lacked the difference in efficiency for Faces compared to Interests as seen in NT participants (greater in the category task, lesser in the exemplar task), and instead processed Faces and Interests in the same way. In scenario (ii), individuals with ASD showed the reverse of the pattern seen in NT participants; for the category task, this means processing Interests more efficiently than Faces, and for the exemplar task, Faces more efficiently than Interests.

For each scenario, the sensitivity was estimated by creating 1,000 surrogate datasets conforming to the hypothesis, and determined how often a significant interaction between diagnosis and category would have been obtained by our analytical procedures. The NT components of the surrogate datasets were generated by standard bootstrapping (i.e., random sampling with replacement) from our sample. The ASD components were also generated by bootstrapping, but the data from each participant were modified to simulate each of the above scenarios. Specifically, in scenario (i), the data for the Faces and Interests trials were randomly interchanged; in scenario (ii), they were systematically swapped. Each of these surrogate datasets was then analyzed in the same way as the actual data, with ANOVAs conducted for the three performance measures (Accuracy, Reaction Time, and Slope) in each of the two tasks. A surrogate dataset was considered to yield a positive result if the *p*-value for the interaction between diagnosis and category was <0.05. Note that, as with the analysis of the actual data, these *p*-values were not corrected for multiple comparisons.

Table 4 reports the results of this analysis. If ASD participants differed from NT participants by having no difference between Faces and Interests (scenario (i), Table 4A), then a significant interaction would be present for at least one of the three performance measures in 987/1,000 of the surrogate datasets in the category task. If the difference between Faces and Interests were reversed (scenario (ii), Table 4B), then a significant interaction would be present for at least one of the three measures in all of the surrogates (1,000/1,000) in the category task. Reaction time was the most sensitive of the three measures. The exemplar task was less sensitive to detecting these two scenarios [171/1,000 for scenario (i), 714/1,000 for scenario (ii)], with accuracy being the most sensitive indicator.

These analyses also provide estimates of power for scenarios in which ASD subjects have a greater performance difference for Interests than Faces in the exemplar task, compared to NT subjects. This is because (as is standard) the ANOVA assumes that main effects are additive, and interactions are multiplicative. Thus, the analysis of Table 4A, in which the interaction is equal to the size of the Interests vs. Faces difference in NT participants, applies not only when the interaction cancels the Interests vs. Faces difference, but also to the case in which it reinforces this difference (and therefore doubles it). As a result, the sensitivity to detect a doubling of the Interests vs. Faces difference in ASD vs. NT participants in the exemplar task is also given by Table 4A, lower row. Similarly, the analysis of Table 4B, in which the interaction is double the size of the Interests vs. Faces difference in NT participants, applies not only when it reverses the Interests vs. Faces difference, but also to the case in which it reinforces this difference (and therefore triples it). This means that the sensitivity to detect a tripling of the Interests vs. Faces difference in ASD vs. NT participants in the exemplar task is also given by Table 4B, lower row. Note, however, that the data (Figure 3) show no suggestion that ASD and NT participants differed in terms of their performance on Interests vs. Faces.

Thus, despite the modest sample size, the category task showed good sensitivity for detecting either of two plausible alterations in the ASD population–likely because the main effect of category was robust (*p* < 0.001 for all three measures). The exemplar task had much lower sensitivity for these specific scenarios, but it could have revealed kinds of differences that the category task overlooked.

## Discussion

The present study sought to examine whether ASD individuals demonstrate a visual processing advantage for unique interests compared to NT controls. We tested this using two visual search tasks: category search and exemplar search. These tasks make different demands on visual processing and tap distinct aspects of early visual search skills: basic classification and subordinate classification. In the exemplar task, RTs were longer for larger array sizes, while in the category task, there was little change in RTs with array size, consistent with prior studies of similar tasks (Jonides and Gleitman, 1972; Smilek et al., 2006). Contrary to our hypotheses that intense interests in ASD may lead to, or result from, differences in early stages of visual processing, there was no evidence of differences between the performance of NT controls and ASD individuals for Interests in either task, as well as no differences for Faces and Houses. Neither children nor adults with ASD demonstrated evidence of visual expertise for their interests relative to age-matched NT controls, even though adults with ASD reported spending more time on their interests than NT adults. The findings are similar to prior work demonstrating no differences in attention (Parsons et al., 2017) or learning (Schuetze et al., 2019) for personalized interests in ASD as compared to NT controls, as well as no differences in visual acuity (Tavassoli et al., 2011). Together the findings suggest that while these search tasks captured low-level visual perceptual differences across key variables (i.e., improved performance with age, increasing RT with array size, predominantly parallel processing for category search), differences in low-level visual perception between ASD and NT participants are relatively minor. While the diagnostic differences in this study are a null finding, this does not rule out the possibility of diagnosis-based differences in visual processing at later stages, or that minor differences in perception are present. Instead, the results demonstrate that the processes required for the current tasks are not large enough to account for the diagnostic behavioral discrepancies between ASD and NT individuals regarding faces and intense interests.

If no causative differences for intense interests occur during early visual perception, then perhaps ASD symptoms relating to intense interests are explained by mechanisms later in the processing stream directly related to reward valuation and executive functioning. Our own work, as well as that of others, suggests that interests are particularly motivating for individuals with ASD. When individuals with ASD observe images of interest they demonstrate greater feelings of pleasure (Sasson et al., 2012). In economic choice paradigms, individuals with ASD value their interests more than a group of NT controls (Watson et al., 2015). Further, regions important for processing arousal, such as the anterior insula (Cascio et al., 2014), as well as reward circuitry, including the dorsal striatum (Kohls et al., 2018), were more sensitive to interests in individuals with ASD than NT controls. Our group has demonstrated that images of interest can interfere with cognitive control in children with ASD but not NT controls (Bos et al., 2019). The present findings suggest that it is likely that intense interests interfere with cognition at the level of arousal and cognitive control in ASD but not visual perception. Future research should seek to directly compare the effects of intense interests on visual perception with the effects on cognitive control. A within-subjects design that utilizes tasks that probe both early visual processing as well as executive functioning may reveal when in the processing stream the differences between ASD and NT individuals occurs.

In both tasks, participants’ accuracy was impacted by category, with highest accuracy for Faces in the category task and for Interests in the exemplar task. In the exemplar task, there was no impact of category on RTs. Overall, the accuracy findings are consistent with our prediction that participants would respond differently to each category of images (Levin et al., 2001). In the exemplar task, there were longer slope values and lower accuracies than in the category search task. This suggests that participants primarily relied on serial processing strategies for the exemplar task and parallel processing strategies in the category task. These behavior patterns are consistent with prior work suggesting that serial processing relies on slower visual strategies compared to parallel processing (Eriksen and Spencer, 1969; Shiffrin and Gardner, 1972). The highly stereotyped nature of responses for both of these visual search skills precludes the need for a within subject paradigm that directly compares performance between these two types of tasks. It is also possible that the exemplar task also had a working memory component, given the need for participants to remember a particular stimulus after a delay. An enhanced working memory for objects of interest may explain why participants were more accurate for interests than for faces. However, there was no significant difference of this effect across diagnostic groups, which is in line with past work that demonstrates working memory differences in ASD only at high working memory loads (Steele et al., 2007).

Given that symptomatology and visual expertise varies with age, participants were divided into two age groups in order to ascertain effects of age on task performance. Consistent with prior visual search studies (Kail, 1991; Donnelly et al., 2007), children had longer reaction times and were less accurate than the adult participants. Also as expected, all participants were faster and more accurate for smaller array sizes in both tasks (Kwak et al., 1991). There were no observed interactions between diagnosis, age, and task performance for Faces, Interests, or Houses. Together these results highlight that both tasks successfully captured early visual search perception in children and adults. While NT participants had significantly higher IQs than ASD participants, this is a well-recognized trait difference in ASD (Richler et al., 2007). In addition, neither VIQ nor NVIQ was related to task performance, demonstrating that the current findings cannot be explained by group IQ differences. Finally, while AQ scores were significantly different between tasks, this is unlikely to explain any results, as the pattern of results across the two tasks was highly similar and all participants were under the cut-off of 32, as suggested by Baron-Cohen et al. (2001).

Interestingly, there was no impact of diagnosis on performance for Faces in either task. These findings were surprising given prior work that has shown general differences in visual processing and visual attention for faces in ASD as compared to NT controls (Boucher et al., 1998; Dalton et al., 2005; Uljarevic and Hamilton, 2013). However, some studies have shown that individuals with ASD are similar to NT controls for certain aspects of low-level face configuration processing. For example, individuals with ASD are susceptible to the face inversion effect (Teunisse and De Gelder, 2003) and are able to detect gaze direction at the same level as NT controls (Gepner et al., 1996). The literature is also mixed on the ability of individuals with ASD to detect facial expressions (Jemel et al., 2006). One possibility is that the visual search paradigm, in which individuals with ASD are known to have an advantage (O’Riordan et al., 2001; Simmons et al., 2009; Kaldy et al., 2016), may have masked the typical processing deficiencies for faces that individuals with ASD exhibit. Future work that examines the confound of enhanced visual search abilities in ASD in domains where individuals with ASD are traditionally impaired, such as face processing, would be helpful in understanding these results. Another possibility is that given the significant heterogeneity associated with ASD (Lord and Jones, 2012), the subset of ASD individuals who completed this task had less severe face processing difficulties than other subgroups of individuals on the spectrum.

There were certain limitations to the present study. First, the interest questionnaire used a different scale for children and adults, making it difficult to combine data across age groups. The child version of the questionnaire also had a limited response range, making it challenging to draw conclusions about the nature of the interests. The images of the interests themselves varied in complexity, which could have affected task performance between participants. However, while we do not quantify the level of complexity for each interest, an examination of the interest list for each group does not suggest a difference in image complexity between groups. More importantly, a complexity difference might lead to a spurious performance difference between the groups, not a lack of difference, as we found. Thus, it is unlikely that image complexity affected the central conclusions of the study. Future studies may wish to systematically manipulate image complexity of both targets and distractors.

The scrambled-distractor paradigm in the category task may be substantially easier than other types of category tasks that use other-category distractors. Although it does not seem that there was a ceiling effect, since there were significant differences in slope across categories, as well as noticeable decreases in accuracy and increases in reaction time across array sizes, future studies may wish to compare category task performance with a scrambled-distractor paradigm to performance with an other-category paradigm. Finally, the number of female participants was too small to assess sex effects in the analyses, which may be informative given the sex imbalance in ASD and the possibility that there is a difference in the effects of interests on behavior across sex (Harrop et al., 2018).

Lastly, due to the modest sample size, a small effect of diagnosis cannot be entirely excluded, especially for the exemplar task, even though our statistics reveal not even a trend in that direction. However, the sample size was adequate to demonstrate dependencies on category and array size, and a power analysis demonstrated that had there been a substantial effect of diagnosis, it would have been detected on at least one measure nearly 100% of the time in the category task, independent from power on the exemplar task. The power analysis used the actual sample sizes for each task, and a hypothetical effect size that was driven by the central question we posed: whether the abnormal interest pattern in ASD subjects could be viewed as merely a consequence of altered search (either a loss of efficient search for faces, or a replacement of efficient search for faces by efficient search for special interests). The reason that high power could be achieved with a relatively small subject pool is that there was relatively little variability of the performance measures within each group (i.e., the error bars in Figures 2, 3 are relatively small.) The exemplar task had much lower power than the category task for scenario (i), which makes sense given that performance differences between faces and houses were only found for the accuracy measure. For scenario (ii), while the power was lower and the direction of the faces-interests performance differential was reversed, a hypothetical effect of diagnosis was still detected 71% of the time.

## Conclusion

In conclusion, individuals with ASD do not show large differences in early visual perception to intense interests compared to NT controls. The findings, while null, suggest that if there are abnormalities in the visual system in individuals with ASD, they are not detectable at the level of visual search with faces or interest images. Further, despite enhanced day-to-day time spent engaging and looking at one’s interest in ASD, there does not seem to be a direct impact of these interests on the early visual system. Together the findings provide insight into the growing body of work to understand the ASD symptoms relating to intense interests.

## Data Availability Statement

The raw data supporting the conclusions of this article can now be found on the project’s OSF page, https://osf.io/rtpwx/ and in the Supplementary Material as Data Sheet S1.

## Ethics Statement

The studies involving human participants were reviewed and approved by Weill Cornell Medicine Institutional Review Board. Written informed consent to participate in this study was provided by the participants’ legal guardian/next of kin.

## Author Contributions

RMJ and BMS contributed to the conception of the study. BMS contributed to data collection and data storage. BMS and JDV contributed to statistical analysis. All authors contributed to the design, read, revised, and approved the submitted manuscript.

## Conflict of Interest

The authors declare that the research was conducted in the absence of any commercial or financial relationships that could be construed as a potential conflict of interest.
