# Supplementary material for: Visual Search for Circumscribed Interests in Autism Is Similar to That of Neurotypical Individuals
Source: Front Psychol. 2020 Oct 21;11:582074. doi: 10.3389/fpsyg.2020.582074 (PMC7640760; doi:10.3389/fpsyg.2020.582074)
Supplement: Supplementary file 2 [file Table_1.docx]

*Table S1A: ASD Interests*

| **Interest** | **Interest Length (1-5)** | **Interest Intensity (1-3)** | **Age** | **Task** |
| --- | --- | --- | --- | --- |
| Super Smash Bros | 4 | 2 | 15.833 | Category, Exemplar |
| Super Smash Bros | 5 | 2 | 25.08 | Exemplar |
| Super Smash Bros | 5 | 2 | 21.33 | Exemplar |
| Super Smash Bros | 5 | 2 | 21.33 | Exemplar |
| Minecraft | 3 | 2 | 10.25 | Category |
| Minecraft | 3 | 2 | 12 | Category |
| Power Rangers | 3 | 2 | 8.17 | Category |
| Power Rangers | NaN | NaN | 20.08 | Category |
| Roblox | 3 | 1 | 11.58 | Exemplar |
| Roblox | 4 | 3 | 12.58 | Exemplar |
| “Bendy and the Ink Machine” Youtube series | 3 | 3 | 9.58 | Category, Exemplar |
| “Extra History” Youtube series | 5 | 2 | 14.75 | Category, Exemplar |
| Broadway | 5 | 2 | 26.33 | Category, Exemplar |
| Cars | 5 | 2 | 10.58 | Category |
| Fantasy | 5 | 2 | 28.33 | Category |
| Great British Bake Off | 5 | 1 | 22.25 | Category, Exemplar |
| Harry Potter | 5 | 1 | 25.83 | Category |
| Legos | 5 | 2 | 12.67 | Exemplar |
| Minion Rush | 3 | 1 | 6 | Category, Exemplar |
| Painting | 5 | 2 | 30.33 | Category, Exemplar |
| Pokemon | 3 | 2 | 11.17 | Exemplar |
| Superheroes | 2 | 2 | 11.33 | Category, Exemplar |
| Traffic Maps | 2 | 2 | 11.67 | Exemplar |
| **Average Children** | 3.43 | 2 |  |  |
| **Average Adults** | 5 | 1.75 |  |  |
| **Average Category** | 4 | 1.86 |  |  |
| **Average Exemplar** | 4 | 1.94 |  |  |

*Table S1B: NT Interests*

| **Interest** | **Interest Length (1-5)** | **Interest Intensity (1-3)** | **Age** | **Task** |
| --- | --- | --- | --- | --- |
| Roblox | 3 | 1 | 8.33 | Category, Exemplar |
| Roblox | 3 | 2 | 9 | Category |
| Roblox | 3 | 1 | 6.08 | Exemplar |
| Harry Potter | 5 | 1 | 19.83 | Category |
| Harry Potter | 5 | 2 | 28.83 | Exemplar |
| Lego World | 5 | 2 | 10.42 | Category, Exemplar |
| Lego World | 3 | 2 | 11.92 | Category, Exemplar |
| Minecraft | 3 | 1 | 10.67 | Category |
| Minecraft | 4 | 1 | 11.5 | Category |
| Buddy the Dog on Youtube | 1 | 3 | 7.75 | Category |
| Chess | 5 | 2 | 26.17 | Exemplar |
| Hiking | 3 | 2 | 22.25 | Exemplar |
| Hockey | 5 | 1 | 12 | Category, Exemplar |
| League of Legends | 4 | 1 | 21.83 | Category |
| Legend of Zelda | 5 | 1 | 27.67 | Category |
| Moana | 3 | 2 | 6.25 | Category |
| Painting | 4 | 1 | 29.33 | Exemplar |
| Paw Patrol | 3 | 2 | 7.75 | Exemplar |
| Pokemon | 5 | 2 | 11.33 | Category |
| Skyrim | 5 | 1 | 21.58 | Exemplar |
| Slime | 3 | 2 | 11.58 | Exemplar |
| The Office | 5 | 1 | 22.17 | Category |
| Wild Kratts | 3 | 1 | 6.33 | Exemplar |
| Yankees | 5 | 2 | 21.42 | Category |
| Yoga | 4 | 1 | 22.75 | Exemplar |
| **Average Children** | 3.36 | 1.64 |  |  |
| **Average Adults** | 4.55 | 1.36 |  |  |
| **Average Category** | 3.93 | 1.53 |  |  |
| **Average Exemplar** | 3.86 | 1.5 |  |  |
